# A novel *MYO6* variant identified in a Chinese family with autosomal dominant non-syndromic hearing loss

**DOI:** 10.1186/s12920-026-02363-0

**Published:** 2026-04-01

**Authors:** Jing Wang, Qing-Wen Zhu, Ai-Ming Cui, Hai-qin Lou

**Affiliations:** 1 Department of Prenatal Screening and Diagnosis Center, Affiliated Maternity and Child Health Care Hospital of Nantong University, Nantong, Jiangsu China; 2 Department of Obstetrics, Affiliated Maternity and Child Health Care Hospital of Nantong University, Nantong, Jiangsu China; 3 Women’s Health Care Department, Affiliated Maternity and Child Health Care Hospital of Nantong University, No. 399 at Shiji Road, Nantong, 226006 Jiangsu Province China

**Keywords:** Autosomal dominant nonsyndromic hearing loss, Whole exome sequencing, MYO6, Novel variants, Molecular dynamics simulation

## Abstract

**Background:**

Hereditary hearing loss (HL) is a highly heterogeneous disorder that follows various inheritance patterns. Variants of *MYO6* gene in DFNA22 are characterised by progressive post-lingual sensorineural HL of varying severity.

**Patients and methods:**

Five-generation Chinese family with autosomal dominant non-syndromic hearing loss (ADNSHL) was enrolled in this study. Whole-exome sequencing (WES) was performed on the proband and her father to screen for causal variants in the genome, whereas intrafamilial co-segregation of the candidate variants in family members was verified using Sanger sequencing. Furthermore, protein modelling and stability analyses were performed to assess the potential pathogenicity of the candidate mutations.

**Results:**

A previously unreported heterozygous missense variant (NM_004999.4:c.2063 A > G, p.Gln688Arg) in exon 20 of *MYO6* using WES and was found to co-segregated with the disease in this family. Molecular dynamics simulations predict that the glutamic acid-to-arginine change in p.(Gln688Arg) alters the normal function, most likely through the altered intermolecular forces of this amino acid with the three nearby polar residues. The structural changes caused by this mutation could potentially affect the myosin ATPase cycle.

**Conclusions:**

We report a novel likely pathogenic missense (c.2063 A > G) variant within of *MYO6* in patients with DFNA22. Our findings expand the variant spectrum of *MYO6* and ADNSHL in Chinese individuals, which will facilitate early clinical genetic diagnosis and accurate genetic counselling of patients.

Hearing loss is one of the most common sensory disorders in humans and its effects are often permanent. Hearing loss is one of the major public health problems facing today’s society. The total number of people with moderate hearing impairment worldwide is approximately 0.5 billion. Among the various causes of deafness, genetic factors account for approximately 60%, and autosomal dominant nonsyndromic hearing loss (ADNSHL) accounts for approximately 20% [1]. The main characteristics of such patients are an obvious family history, clinical heterogeneity and progressive post-speech hearing loss [2], which is difficult to detect early in clinic owing to its occult onset and slow progression. Mutations in a single gene with a specific function in the inner ear are the root cause of the disease [3].

Human hearing perception relies on the ability of the inner ear to converts mechanical energy into electrical impulses, and hair cells located in the organ of Corti within the cochlea play an important role in this process [4]. Stereocilia, a cluster of parallel filamentous actin (F-actin)-filled stereocilia on the apical specialisation of the hair cells that are arranged in a staircase-like fashion and inserted into the cuticular plate, shows a tapering morphology, that plays an important role in mechano-electrical transduction [5]. Several proteins related to the function of the basal region of the static cilia have been shown to affect cochlear tuning and sensitivity by influencing the density of filamentous actin and the morphology of the stereocilia root, causing late-onset hearing loss [6, 7].

Myosin VI (MYO6) is a molecular motor containing an N-terminal domain that enables translocation along actin filaments, a regulatory light chain-binding region, a coiled coil domain that mediates its dimerisation, and a C-terminal tail region that regulates cytoskeletal track dynamics [8]. As an unconventional myosin, MYO6 is expressed in the cuticular plates, pericuticular necklaces, and cytoplasm of the OHCs and IHCs of both inner and outer hair cells and plays an important role in cargo transportation and stereocilia anchors [9]. Mutations in human *MYO6* can cause autosomal recessive DFNB37 and autosomal dominant DFNA22 non-syndromic hearing loss (NSHL; https://omim.org/entry/600970). Mutations in *MYO6* are widely recognised as one of the main causes of NSHL [10]. To date, nearly 100 pathogenic or potentially pathogenic variants of *MYO6* have been associated with HL (http://deafnessvariationdatabase.org). With the rapid development of next-generation sequencing technologies, new mutations are constantly being identified. However, subsequent studies on genotype-phenotype associations are expensive and time-consuming. Thus, understanding the molecular mechanisms by which mutations in MYO6 cause disease has become a challenge for researchers.

With the rapid development of computer technology and simulation algorithms, the impact of molecular dynamics (MD) simulations have become an important means of studying the dynamic behaviour of biological macromolecules such as proteins and DNA [11, 12]. By simulating the motion state of biological macromolecules in three-dimensional space, the position and motion state of each atom can be recorded with very fine time resolution, which aids in understanding the structure-function relationship of proteins at the atomic level [13, 14].

In this study, we identified a heterozygous missense variant in *MYO6* using whole exome sequencing (WES) in a large Chinese ADNSHL family with six affected members and confirmed the co-isolation of the variant in more family members. We also used homology modelling and molecular dynamics simulations to compare the physiological functions of mutant protein and wild-type proteins, providing a theoretical basis for exploring the association between mutations and the pathogenesis of deafness.

## Materials and methods

### Subjects and clinical evaluation

An extended ADNSHL Chinese family spanning five generations was found in the ear clinic of Affiliated Maternity and Child Health Care Hospital of Nantong University, and detailed interviews were conducted with the proband and their families to understand their medical and family history, including age of onset, hearing loss (degree and evolution trend of hearing loss), accompanying symptoms (tinnitus, vertigo, headache, etc.), ototoxic drug use history, craniocerebral trauma history, noise exposure history, etc. High-resolution computed tomography (HRCT) of the temporal bones was performed to exclude inner ear malformations. All patients underwent clinical audiological evaluation, including auditory brainstem response audiometry (ABR), multipleauditory steady-state evoked responses (ASSR), transient-voked otoacoustic emissions (TEOAE), acoustic immittance, and pure tone audiometry (PTA). The severity of hearing impairment was defined as follows: normal hearing (< 20 dB), mild hearing loss (20–35 dB), moderate hearing loss (35–50 dB), moderate-to-severe hearing loss (50–65 dB), and severe-to-profound hearing loss (> 65 dB).

### Whole-exome sequencing

Peripheral blood (2 ml) was collected from each patient into a tube containing disodium ethylenediaminetetraacetic acid (EDTA-Na_2_). A blood genomic DNA extraction kit (Tiangen Biochemical Technology Co. Ltd., Beijing, China) was used according to the manufacturer’s protocol.

Genomic DNA (200 ng) was enzymatically fragmented into approximately 200 bp fragments, subjected to end repair, followed by A-tailing at the 3’ end, and subsequently ligated with adapters for PCR amplification to get the amplified library. The library (750 ng) was mixed with the hybridisation reaction mixture and added to a PCR tube for hybridisation. After washing, eluted DNA was mixed with a combination of amplification enzymes and primers. DNA fragment size was detected using an Agilent 2100 Bioanalyzer (Agilent, Santa Clara, CA, USA), and was required to be within the range of 150–500 bp, with the major peak at approximately 260–300 bp, to proceed to the next experimental step. Exome Capture was performed using the Basecare Medical Probes Reagent (Agilent, Santa Clara, CA, USA). The concentration and fragment size of the post-capture library were assessed using a Qubit dsDNA HS Assay Kit and Agilent 2100 Bioanalyzer, respectively. Finally, high-throughput sequencing was performed on the MGISEQ-2000 platform (BGI, Shenzhen, Guangdong, China) in the paired-end (PE) 150-base pair mode to obtain the raw sequencing data.

### Data processing

Raw sequencing data were processed using Trimmomatic to remove adapters and low-quality sequences. BWA software was used to align the sequencing data to the GRCh37/hg19 reference genome (University of California at Santa Cruz), retaining only variants located within the coding sequences or splice site regions. Genome Analysis Toolkit 4 (GATK4) software was used to mark duplicate sequences, recalibrate quality scores, and identifying variants. SNP and InDel variants were filtered and selected based on sequence depth and mutation quality. ANNOVAR was employed to annotate the variants, including population frequency databases; such as 1000 Genomes, gnomAD, ExAC, and ESP. Deep intronic variations were excluded.

Phenolysers are used to associate patient phenotypes with genes to further refine variant selection. Pathogenicity was determined according to the expert guidelines in the American College of Medical Genetics and Genomics (ACMG) and the Association for Molecular Pathology (AMP) Guidelines(2015 ACMG/AMP guidelines) [15] and Expert specification of the ACMG/AMP variant interpretation guidelines (2018 HL-EP guidelines) for genetic hearing loss [16]. To reliably detect copy number variations (CNVs) from WES, Genome Analysis Toolkit (GATK 4).GermlineCNVCaller was performed given read-depth which was calculated by GATK CollectReadCounts. Finally, the log2 ratio of the read depth on each exon was estimated to infer discrete copy number segments.

### Sanger sequencing verification of the *MYO6* gene

Sanger sequencing was performed to verify a novel mutation (c.2063 A > G) in exon 20 of *MYO6*. Primers were designed according to the *MYO6* sequence (GenBank NM_004999.4) using Primer 3 software (http://primer3.ut.ee/) as follows: forward, 5′-TGCTCCCAGCCTTGAGTTCT‐3′; reverse, 5′‐AGATCCAGTTTTGCTGTTAGAGAGTT‐3′. The PCR products were sequenced using an ABI 3500 Genetic Analyser (Applied Biosystems, Foster City, CA, USA).

### In silico analysis

The pathogenecity of missense variants was predicted using Revel (an ensemble method for individually predicting the pathogenicity of missense variants using the following tools: MutPred, FATHMM, VEST, PolyPhen, SIFT, PROVEAN, MutationAssessor, MutationTaster, LRT, GERP, SiPhy, phyloP, and phastCons; with a cutoff value of > 0.7). The MEGA program (version 11) was used to align *MYO6* sequences from different species using default parameters and viewed using the Jalview program(Version: 2.11.3.3, University of Dundee, Scotland, UK).

### Molecular dynamics simulations

The structure of the MYO6 protein was obtained from the AlphaFold database (https://alphafold.ebi.ac.uk/). Based on the Maestro academic version, we obtained the MYO6_Q688R mutant and subsequently performed all-atom molecular dynamics simulations using the AMBER 18 software1. Prior to the MD simulations, the AMBER force field ff14SB2 was applied to describe the proteins. Subsequently, complex system was immersed into a rectangular periodic box of pre-equilibrated TIP3P water with at least 10 Å distance around the complexes. Finally, appropriate numbers of sodium counter ions were added to maintain the electroneutrality of the simulated system.

For each simulation, a sophisticated protocol (minimisation, heating, equilibration and production) was followed. Initially, water molecules were minimised through 2500 steps of steepest descent, followed by 2500 steps of conjugate gradient, whereas proteins were kept at the same position except for the hydrogen atoms. The same minimisation protocol was applied to optimise the side chains. Finally, the entire system was relaxed for 5000 steps without any restraints. After energy minimisation, each system was gradually heated to a constant volume from 0 K to 300 K over a coupling time of 100 ps with position restraints. To accommodate the solvent density, the entire system was equilibrated for 100 ps at a constant pressure of 1 bar. Subsequently, another 100 ps pre-equilibration was performed for pressure relaxation with a weak restraint on the protein backbone. Subsequently, a 100 ns MD simulation was conducted for each system to produce the trajectories. During the MD simulations, periodic boundary conditions were employed and the direct space interaction was calculated using the particle mesh Ewald (PME) method with a long-range electrostatic interaction [17]. All bonds involving hydrogen atoms were.

constrained using the SHAKE algorithm [18], which allowed an integration time step of 2 fs. Finally, the trajectory was saved every 20 ps for subsequent analysis.

## Results

### Clinical characteristics

**Fig. 1:**
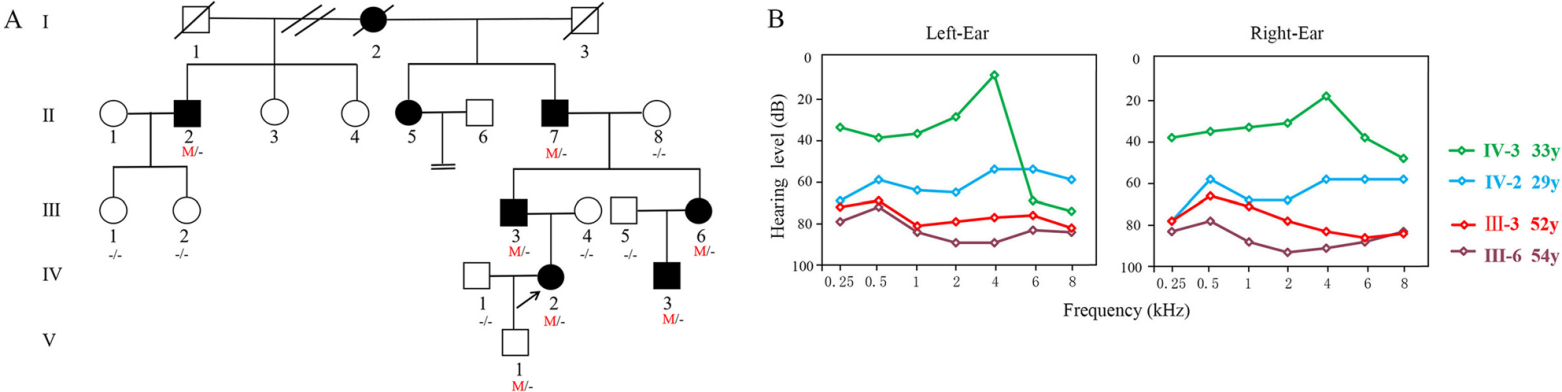
Family Pedigree and audiograms with autosomal dominant hearing loss. **A** Filled symbols indicate the individuals suffer from hearing impairment.and open symbols indicate unaffected individuals. The black arrow indicates the proband (Ⅲ:2). **B** Audiograms of patients with MYO6-associated hearing loss. M, c.2063 A > G; -, wild type

As can be seen from the family pedigree (Fig. 1A), the disease appears in every generation, regardless of sex. These characteristics are consistent with the genetic law of autosomal dominant inheritance. The family included seven individuals carrying the mutation in five generations, and the symptoms of six affected adults were mainly varying degrees of hearing loss after adolescence, and normal intellectual and speech function (blood samples and clinical data was not obtained from Ⅱ5); Ⅴ-1, a 6-month-old infant, passed the neonatal hearing diagnostic test. Family members had no history of noise exposure or ototoxic drug use. Specialist examination revealed that the auricle and external auditory canal were not deformed and the tympanic membrane was intact. The hearing curve of patients with hearing loss in this family was consistent with the loss of bone conduction, both of which are symmetrical binaural sensorineural hearing losses, but the age of onset (range, 12–26 years) varied significantly. The current audiogram does not reflect the progression of hearing loss; however the hearing curve of patients with mild hearing loss shows a steep decline with high-frequency hearing loss, whereas those with moderate and severe hearing loss show a flat audiogram (Fig. 1B). Clinical evaluation of the proband showed no abnormalities in the cardiac structure or function. An eye examination demonstrated 5.50 diopters in the right eye and 6.00 diopters in the left eye. She also had non-alcoholic fatty liver disease. Assessments of the six affected adults revealed no other clinical symptoms or signs except for Ⅳ-3 who had colour vision deficiency (Table 1).

**Table 1:** Summary of the phenotypic information of the family members

Subjects	Gender	Age(years)		PTA (dBHL)		Audiogram shape	Degree of HL	Clincal evaluation
		At onset	At testing	Left	Right			
Ⅱ2	Male	-	75	-	-	-	-	-
Ⅱ7	Male	-	73	-	-	-	-	-
Ⅲ3	Male	18	52	78	79	flat	severe to profound	NA
Ⅲ6	Female	13	54	88	90	flat	severe to profound	NA
Ⅳ2	Female	12	29	63	66	flat	moderate to severe	short-sightedness , non-alcoholic fatty liver disease.
Ⅳ3	Male	26	33	42	39	down-sloping	moderate	colour vision deficiency
Ⅴ1	Male	NA	1	normal	normal	normal	normal	NA

NA, normal; -, not evaluated

### Genetic analysis

Whole-exome sequencing was performed on the Ⅲ-3 and Ⅳ2 to investigate the candidate deafness genes. An average of 25 billion base sequences were produced per individual using a paired-end 150-bp (PE150) sequencing platform. After filtering low-quality (Q < 20) reads and eliminating adapter sequences, the clean reads were aligned to the human genome reference assembly (Build 37.1, hg19). Approximately 99.68%-99.72% of the clean reads were uniquely aligned to the reference genome and more than 80% were located in the target region. In this case, greater than 250X average depth was in the target regions and more than 98% of target regions showed read depths ≥ 30X, which satisfied the requirements for calling single-nucleotide polymorphisms (SNPs) and indels. Next, a stepwise approach was used to screen for pathogenic variants in the patients. (1) Various databases (1000 Genome Project, gnomAD version 2.1.1, EXAC, ESP6500, and an in-house database) were used for basic MAF filtration, and all alleles with minor allele frequencies lower than 0.01 were filtered. (2) Variants found in all affected individuals. (3) Candidate variants (SNV, Indel, CNV) were classified according to the international guidelines of the ACMG using related scientific literature and the disease databases OMIM, Clinvar and HGMD. (4) The rare exome variant ensemble learner (REVEL) software was used to predict the pathogenicity of missense variants.

### In silico analysis

**Fig. 2:**
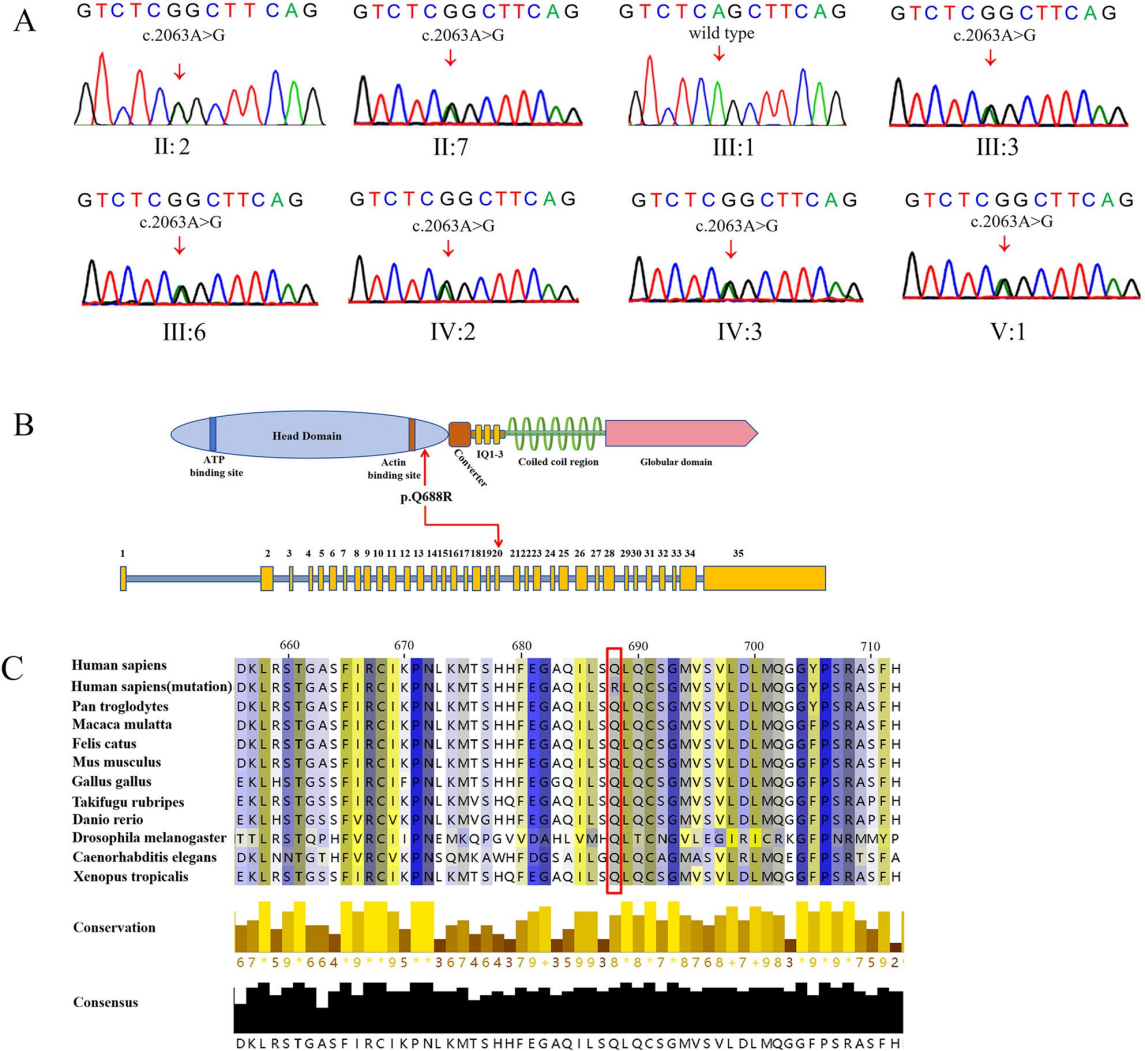
Mutational analysis of the c.2063 A > G *MYO6* variant. **A** Sanger sequencing of the heterozygous missense c.2063 A > G mutation and wild-type control in exon 20 of *MYO6*. **B** Location of variants on *MYO6* gene with corresponding amino-acid location; *MYO6* gene NCBI reference sequence of NG_009934.2, transcript reference of NM_004999.4, and protein reference of NP_004990.3. **C** Protein alignment showing that MYO6 p.Q688R occurs at evolutionarily conserved amino acids across the 10 species. Multiple alignments were performed using MUSCLE implemented in JalView

We identified one variant with an A-to-G substitution at nucleotide position 2063 (c.2063 A > G) in exon 20 of the *MYO6* gene, which was confirmed by Sanger sequencing (Fig. 2A and B). This variant has not been reported in the Human Genetic Variation Database or gnomAD database. It converts the highly conserved Gln to Arg at amino acid position 688 (p.Gln688Arg; Fig. 2C). The results of the prediction programs showed that the variation had a deleterious effect, with a REVEL score of 0.95. The mutation segregated with affected family members but not with unaffected family members, and was not observed in control individuals. Based on the ACMG guidelines, the variant can be categorized as likely pathogenic (PM2+PP1_strong+PP3). Moreover, we did not identify potentially pathogenic mutations or CNV in any of the other genes implicated in the proband and her father.

### Molecule dynamics analysis

**Fig. 3:**
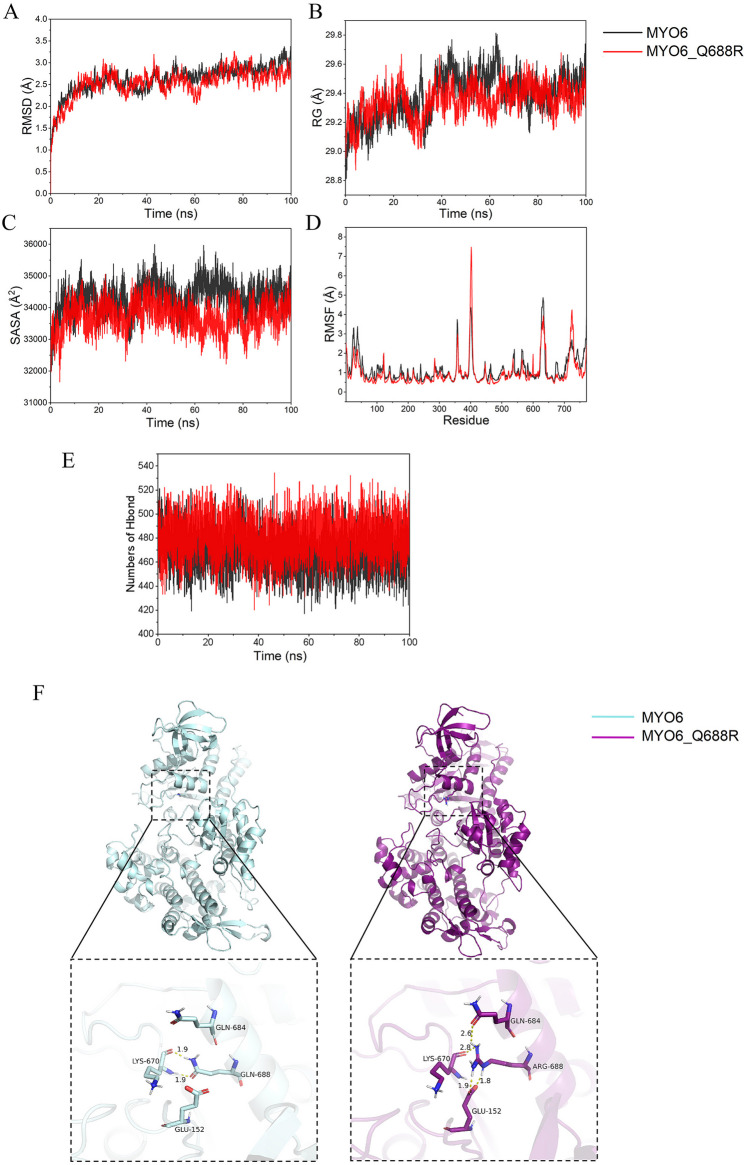
The dynamic characteristics of proteins after the combination of different small molecules: (**A**) Protein RMSD changes over time; (**B**) Protein RG changes over time; (**C**) Protein SASA changes over time; (**D**) Protein RMSF in different complexes. **E** The number of hydrogen bonds formed within the protein over time during the molecular dynamic simulation was compared between the mutant and wild-type. **F** Molecular protein structure of the motor domain of MYO6 showing p.Q688 and the p.Q688R variant

The root-mean-square deviation (RMSD) in molecular dynamics simulations reflects the motion of the complex (Fig. 3). As shown in Fig. 3A, the p.Q688R variant reached a converged state at approximately 20 ns in the simulation and exhibited stable fluctuations at approximately 2.5 Å, and the average RMSD value of was 2.53, which is lower than wild-type MYO6 protein (RMSD = 2.61Å), indicating that the mutant protein is more stable. Similarly, The mutation protein was more compact with an Rg score of 29.27 Å than the wild-type protein having an Rg score of 29.36 Å, indicating an increase in the globularity of protein (Fig. 3B). In the solvent-accessible surface area (SASA) data, while the average SASA value for the wild-type MYO6 protein was 34,297 Å2, that for the mutant protein was 33,562 Å2 (Fig. 3C), indicating that the surface interactions of the mutant protein were stronger and more stable, affecting the likelihood of interaction with other molecules. Finally, from the root-mean-square fluctuation (RMSF) plot (Fig. 3D), we observed that the mutant had lower overall RMSF values than the wild-type protein, whereas the RMSF of the mutant arginine residues was smaller than that of wild-type glutamine residues (0.59 vs. 0.64), indicating that the mutation affected the residual flexibility at position 688 along with the overall flexibility of the protein. Hydrogen bonds within proteins play an important role in maintaining the internal interactions. A higher number of hydrogen bonds indicates stronger internal interactions and better folding. As shown in the Fig. 3E, the mutant had more hydrogen bonds than the wild type (WT), indicating that the mutant had more internal interactions and that it folded better. The 688th amino acid of the WT is Gln, which can form two hydrogen bonds with Lys-670. However, in the mutant, the 688th position is changed to Arg, which not only forms a hydrogen bond with Lys-670 but also interacts with Gln-684 and Glu-152, forming a total of two salt bridges (Fig. 3F).

## Discussion

Mutations in human *MYO6* are involved in recessive DFNB37 and dominant DFNA22 non-syndromic hearing loss. In recent years, advances in genetic diagnostic techniques have uncovered a large number of novel variants to explain sequence variation in hearing loss genes according to the standardization of variant interpretation in the ACMG guidelines, which has further enriched the phenotypic spectrum of *MYO6* [19]. In this study, we identified a novel missense mutation in *MYO6*, which has never been reported before and further silicon studies were performed to determine the potential effect of the missense variant on MYO6 function.

In this study, we identified a novel missense mutation (c.2063A > G; p. Q688R) in the head domain of MYO6 as the cause of ADNSHL in a family with an age of onset ranging cases ranged from 12 years to 26 years. A 6-month-old infant (Ⅴ1) in a family with normal audiometry carried the same mutation. Due to the patient’ s young age, it cannot be ruled out that hearing loss may occur later in life, as was the case with other family members. Except for Ⅳ-3, all other patients in this family had a post-lingual early onset (12–18 years old), and the audiogram revealed a symmetrical, bilateral, full-frequency, flat contour, and moderate-to-severe sensorineural pattern. In patient Ⅳ-3 (7 years of medical history), a very steep drop in audiometric configuration was recorded at higher frequencies, showing mild HL between 250 and 2,000 Hz that increased to severe HL at 8,000 Hz. Our results suggest that the severity of the hearing loss is related to the length of time it has occurred, it develops as a result of prolonged abnormal *MYO6* function, and that the *MYO6*-mediated variable exhibiting variable expressivity of HL is clinically common. Zubair et al. reported a patient with the homozygous variants p.R1166X presented with bilateral, profound, sensorineural, congenital hearing loss, whereas heterozygous carriers were normal [20]. However, this p.R1166X variant was reported to cause mild HL in five unrelated families in a large-scale genetic analysis of Japanese patients with HL [21].The phenotypic differences were observed in heterozygous or homozygous individuals paired with *MYO6* c.897G > T variants [22]. Failure to perform an audiometric evaluation may result in heterozygous individuals in the DFNB37 family at the risk of HL being overlooked. Stereocilia are mechanically sensitive hair cell regions that rotate around a cone during mechanical stimulation. Therefore, the conical shape of stereocilia inserted into the stratum corneum is important for functional maintenance [23]. As a cargo transporter, MYO6 can move critical regulatory components such as chloride intracellular channel 5, protein tyrosine phosphatase receptor Q, radixin and taperin proteins to the bases of the stereocilia of the cuticular plate to participate in the maintenance of taper [24–26]. Mutations in MYO6 proteins with their motion and ability to combine and transport tapering proteins, resulting in decreased taper-specific proteins at the base of the stereocilium, thus leading to hearing deficits caused by disorganised hair bundles, vertical cilia fusion, and taper loss [27]. A heterozygous *Myo6*^*em1Tmims/+*^ mouse model was consturcted to evaluate the *MYO6* haploinsufficiency effects on hearing ability. The results confirmed that *MYO6* haploinsufficiency influenced several hearing phenotypes, including mild HL, stereocilia disorganisation, and age-related IHC ribbon loss [28]. Hearing loss phenotypes caused by haploinsufficiency can account for stoichiometric imbalances in macromolecular complexes or cellular networks [29]. It is noted that hearing loss progressed more rapidly in female *MYO6* +/- heterozygous mice than in male mice were observed in previous studies [28]. Researchers believe that the hearing levels of female C57BL/6 mice are slightly lower than those of males mice, although this difference is even more pronounced because of the *MYO6* genotype effect [30, 31]. Schultz et al.suggests that the mutation p.V586M in the plasma membrane calcium pump, PMCA2, encoded by ATP2B2, may modify the severity of hearing loss caused by *MYO6* mutations [32]. Notably, associations between sex age of onset, and severity of deafness were also observed in this study. Based on these results, we suggest that the weak pathogenicity of *MYO6* haploinsufficiency, epigenetic regulation of gene expression and other genetic variants with additive or epistatic modifier effects may be associated with *MYO6*-mediated variable exhibit variable expressivity of hearing impairments [33–35].

To date, more than half of all pathogenic/likely pathogenic missense mutations in *MYO6* have been found in the motor head domain (http://deafnessvariationdatabase.org), indicating the importance of the myosin motor domain in MYO6 executive function (Table 2) [21, 29, 36–46]. For example, in 2001, Sanggaard reported the first family with ADNSHL that is characterised by progressive postlingual onset due to *MYO6* missense mutation (p.C442Y) [44]. The mutation is located near the switch II loop, which is known to affect ADP dissociation from actomyosin, significantly increases the dissociation of ADP from both motor Myosin VI and actin filaments, and disturbs the processive movement of myosin VI on actin filaments [47]. During evolution, the spatial structures of proteins have become more conserved than amino acid sequences. To investigate the structural impact of the point mutation p.Q688R on the *MYO6* gene, a total 100 ns molecular dynamics simulation was performed to determine the time-dependent behaviour of the wild-type and mutant proteins. This investigation revealed that the mutation resulted in increased protein stability, densification and hydrogen bond formation, which are important factors in protein activity. The myosin head is an ATPase, the major components of which is composed of three loop structures called the P-loop (GESGAGKT, amino acids 151–158), switch I loop and switch II loop, which play important roles in converting chemical energy into shrinking mechanical energy [48, 49]. Modelling of the motor head area showed that unlike wild-type Gln-688, which forms two hydrogen bonds with Lys-670. In the mutant, Arg-688 forms a hydrogen bond and two salt bridges with the carbonyl oxygen of Lys-670 and the anionic carboxylate (RCOO-) of Glu-152, respectively. Recent studies have suggested that the interaction between the backbone carbonyl oxygen of Ser-153 and the side chain amide group of Lys-670 on the switch-II loop modulate the stability of specific P-loop states, which is important for maintaining MYO6 high-duty-ratio motor performance characteristics [50, 51]. We reasoned that the mutation changes the intermolecular interaction force with the Switch II loop and P-loop simultaneously, affects the nucleotide compatibility of the P-loop conformations and the release of Pi, and alters ADP dissociation rates from actomyosin, thus interfering with the myosin ATPase cycle process and inhibiting the motor action of the protein, leading to deafness.

**Table 2:** Summary of all known *MYO6* pathogenic/likely pathogenic missense variants and their hearing loss phenotypes

Nucleotide Change	Amino Acid Change	Exon	Audiogram profle	Phenotype	Clinical evaluation	References
c.178G > C	p.Glu60Gln	Exon3	-	AR, severe to profound	NA	Alkowari et al. 2017 [29]
c.473 C > G	p.Thr158Arg	Exon6	flat/ U-shaped	moderate	-	Kim et al.2018 [36]
c.584 C > A	p.Ala195Glu	Exon8	high frequency impairment	moderate	-	Seco et al. 2017 [37]
c.599 A > G	p.Asn200Ser	Exon8	-	moderate	NA	Morgan et al. 2019 [38]
c.614G > A	p.Arg205Gln	Exon8	U-shaped or flat	mild to moderate	NA	Kwon et al. 2014 [39]
c.622 A > G	p.Lys208Glu	Exon8	flat–sloping	severity of HL associated with age	NA	Tian et al. 2018 [40]
c.647 A > T	p.Glu216Val	Exon8	-	AR, severe to profound	NA	Ahmed et al. 2003 [41]
c.667G > A	p.Gly223Arg	Exon9	down-sloping	moderate	-	Kim et al. 2018 [36]
c.734 A > G	p.Tyr245Cys	Exon9	-	-	-	Yang et al. 2013 [42]
c.737 A > G	p.His246Arg	Exon9	variable	rates of HL progression are highly variable	familial hypertrophic cardiomyopathy	Mohiddin et al. 2004 [43]
c.897G > T	p.Glu299Asp	Exon10	-	moderate	-	OKA et al. 2020 [21]
c.1015 C > T	p.Arg339Trp	Exon11	-	moderate	-	Yang et al. 2013 [42]
c.1322 A > C	p.Gln441Pro	Exon13	Cookie–bite	moderate	NA	Frohne et al. 2021 [45]
c.1325G > A	p.Cys442Tyr	Exon13	down-sloping	moderate to profound	NA	Melchionda et al. 2001 [44]
c.1681 A > G	p.Arg561Gly	Exon17	-	severe	Tinnitus	Ji et al. 2014 [46]

NA, normal; -, not provided

In conclusion, we report the identification of an exon-pathogenic *MYO6* c.2063 A > G gene variant found in a Chinese family with ADNSHL, provide a classification of the pathogenicity of the variants, and describe the characteristic clinical features of patients with hearing loss. It is not only conducive to the early detection of patients with atypical clinical symptoms for timely intervention, but also provides a basis for counselling on and preventing the inheritance of deafness.

## Data Availability

Data is provided within the manuscript or supplementary information files.
